# The Effect of Cell Morphology on the Permeability of the Nuclear Envelope to Diffusive Factors

**DOI:** 10.3389/fphys.2018.00925

**Published:** 2018-07-13

**Authors:** Alberto García-González, Emanuela Jacchetti, Roberto Marotta, Marta Tunesi, José F. Rodríguez Matas, Manuela T. Raimondi

**Affiliations:** ^1^Laboratori de Càlcul Numèric, E.T.S. de Ingenieros de Caminos, Canales y Puertos, Universitat Politècnica de Catalunya – (UPC BarcelonaTech), Barcelona, Spain; ^2^Department of Chemistry, Materials and Chemical Engineeering “Giulio Natta, ” Politecnico di Milano, Milan, Italy; ^3^Electron Microscopy Facility, Istituto Italiano di Tecnologia, Genoa, Italy; ^4^Unità di Ricerca Consorzio INSTM, Politecnico di Milano, Milan, Italy

**Keywords:** nuclear pore complex, passive diffusion, nuclear envelope permeability, stem cell differentiation, finite element modeling, scanning transmission electron microscopy, confocal microscopy

## Abstract

A recent advance in understanding stem cell differentiation is that the cell is able to translate its morphology, i.e., roundish or spread, into a fate decision. We hypothesize that strain states in the nuclear envelope (NE) cause changes in the structure of the nuclear pore complexes. This induces significant changes in the NE's permeability to the traffic of the transcription factors involved in stem cell differentiation which are imported into the nucleus by passive diffusion. To demonstrate this, we set up a numerical model of the transport of diffusive molecules through the nuclear pore complex (NPC), on the basis of the NPC deformation. We then compared the prediction of the model for two different cell configurations with roundish and spread nuclear topologies with those measured on cells cultured in both configurations. To measure the geometrical features of the NPC, using electron tomography we reconstructed three-dimensional portions of the envelope of cells cultured in both configurations. We found non-significant differences in both the shape and size of the transmembrane ring of single pores with envelope deformation. In the numerical model, we thus assumed that the changes in pore complex permeability, caused by the envelope strains, are due to variations in the opening configuration of the nuclear basket, which in turn modifies the porosity of the pore complex mainly on its nuclear side. To validate the model, we cultured cells on a substrate shaped as a spatial micro-grid, called the “nichoid,” which is nanoengineered by two-photon laser polymerization, and induces a roundish nuclear configuration in cells adhering to the nichoid grid, and a spread configuration in cells adhering to the flat substrate surrounding the grid. We then measured the diffusion through the nuclear envelope of an inert green-fluorescent protein, by fluorescence recovery after photobleaching (FRAP). Finally, we compared the diffusion times predicted by the numerical model for roundish vs. spread cells, with the measured times. Our data show that cell stretching modulates the characteristic time needed for the nuclear import of a small inert molecule, GFP, and the model predicts a faster import of diffusive molecules in the spread compared to roundish cells.

## Introduction

The mechanobiological cues guiding stem cell fate are currently being intensely explored *in vivo* (Rompolas et al., 2013) and *in vitro* (Nava et al., 2012). *In vitro*, they can be modulated through substrate stiffness, surface nanotopography, microgeometry, and extracellular forces. For example, the culture of mesenchymal stem cells (MSCs) on substrates with tuned elasticity (Swift et al., 2013), or with a size and geometry constraint (Nathan et al., 2011; Tseng et al., 2012), results in an alteration in cell spreading, leading to major remodeling of the cellular cytoskeleton. This remodeling, in turn, alters the nuclear shape, mediated by the traction transmitted to the nucleus by the filamentous actin cytoskeleton (Badique et al., 2013). However, exactly how alterations in nuclear shape are transduced into stem cell fate are unknown.

Here, we hypothesize that strain states in the nuclear envelope (NE) cause changes in the structure of the nuclear pore complex (NPC). This would lead to a significant change in the permeability of the nuclear envelope to the traffic of those transcription factors involved in stem cell differentiation which are very small and thus imported in the nucleus through the NPCs by simple passive diffusion. The molecular weight of these diffusive molecules has been estimated to be lower than 40 kDa (Paine et al., 1975; Ribbeck and Görlich, 2001) but can reach dimensions up to 70 kDa (Wei et al., 2003; Cardarelli and Gratton, 2010; Bizzarri et al., 2012).

**Figure 1:**
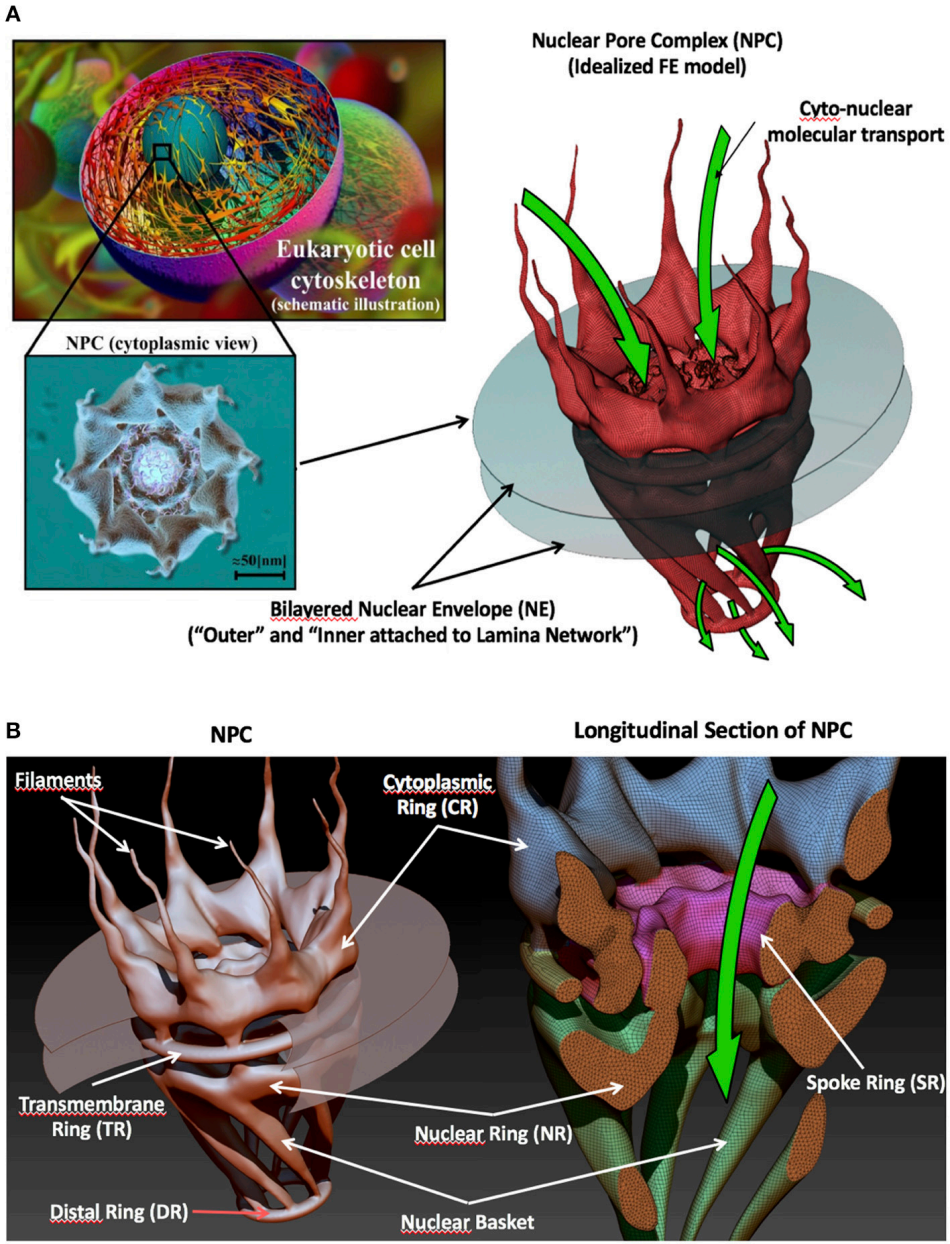
The nuclear pore complex. **(A)** From micro (STEM cell) to nanoscale (nuclear pore complex structure). **(B)** Main sub-structural groups (rings, filaments, and basket) that make up an NPC. (Reproduced from Garcia et al., 2016 with permission from the Royal Society of Chemistry).

The multiprotein structure of a NPC is detailed in Figure 1. NPCs are a substructure assembly composed of several coaxial rings and 8-fold rotational (Goldberg and Allen, 1996; Beck et al., 2004; Löschberger et al., 2012) symmetrical structures named according to their spatial location: (1) The cytoplasmic ring (CR) and filaments in the cytoplasmic side; (2) The spoke ring (SR) and transmembrane ring, which provide stiffness and stability to the complete NPC, in the nuclear envelop; (3) The nuclear ring (NR), which is attached to the lamina, the nuclear basket, and the distal ring (DR) in the nuclear side. For a detailed description of the structure of the NPC, see the review paper (Garcia et al., 2016).

NPCs pose efficient barriers to big inert objects (Mohr et al., 2009) and regulate the protein translocation between the cytoplasm and cell nucleus, thus suppressing an intermixing of the contents of the two compartments in order to control cell life and regulate gene expression, as in cell differentiation. Small proteins and molecules can pass unassisted through the NPC by passive diffusion. This translocation process becomes increasingly restricted as the particle size increases (Paine et al., 1975; Wei et al., 2003). Passive diffusion becomes very inefficient approaching an upper molecular weight limit of around 40–70 kDa. Thus, larger proteins are let into the nucleus by a NPC selective receptor on the FG-domain, which recognizes a specific import motif (called the nuclear localization signal) expressed by the cargos. This process of protein translocation, named facilitated translocation, is often associated with an input of metabolic energy, thus enabling transport also against a concentration gradient (Paine et al., 1975; Ribbeck and Görlich, 2001; Naim et al., 2007).

According to the basic principles of mass transport, the nuclear flux of small transcription factors occurring by passive diffusion should be proportional to their concentration gradient across the NPC, by a coefficient related to the dimension of the pore lumen. Variable diameters have been observed in the NPC, likely made possible by large-scale rearrangements of double-ring protein subcomplexes (Bui et al., 2013). Such large-scale rearrangements are now believed to be biologically significant only for the transport of huge macromolecular cargoes.

To the best of our knowledge, no one has yet hypothesized a role for the pore dimensional variations in regulating the purely diffusive nuclear fluxes of signaling molecules, such as transcription factors, including those involved in stem cell differentiation. This work defines one of these mechanisms using an advanced mechanobiology model based on the integration of a computational model of protein nuclear diffusion with nuclear deformation, with direct measurements on the cells of nuclear import flows of small diffusive proteins.

Computational modeling of nuclear diffusion-deformation phenomena entails coupling structural mechanics models for the NE and NPC with diffusion equations for the transcription factors, which is an essentially unexplored field. Few published examples of numerical simulations address the mechanics of the NPC and its effect on nucleocytoplasmic transport (reviewed in Garcia et al., 2016). At the cell scale, our group developed a finite element simulation of passive diffusive fluxes from the cytoplasm to the nucleus, accounting for nuclear deformation (Nava et al., 2015a). This model coupled nuclear diffusion with local NE deformation in transient conditions, through a strain-dependent diffusion coefficient. At the nanoscale, numerical simulations based on molecular dynamics predicted a cargo trajectory through an NPC by interaction with the FG-domain of an NPC selective receptor (Moussavi-Baygi et al., 2011). This model also supports the hypothesis that the mechanical response of the NPC may affect the diffusion of cargos and smaller molecules through the nuclear pore.

A major challenge in calibrating these numerical models is the direct measurement of small diffusive proteins in cells of the nuclear import flows. The study of protein mobility or translocation of protein between different compartments of live cells (such as nucleocytoplasmic translocation) was made possible by the discovery and development of fluorescent proteins (FPs) (Chalfie, 1994; Tsien, 1998). FPs are a class of genetically encodable proteins derived from sea organisms and, in particular, from the jellyfish *Aequorea victoria*.

Using molecular biology techniques and commercial scanning microscopes, FPs can be tagged to any protein of interest. In addition, fluorescent microscopy can visualize, localize and track proteins in live cells and also reveal the extensive networks of protein-protein interactions that regulate cell processes (Lippincott-Schwartz et al., 2003). Fluorescence recovery after photobleaching (FRAP) is particularly useful in assessing the dynamic and biochemical properties of intracellular proteins in a single or multiple cell compartment (Sprague and McNally, 2005). FRAP was originally conceived in 1974 by Peters et al. (1974) and is very useful for studying protein mobility because it is only based on the change in optical properties, whereas the dynamics and biochemistry of the molecules of interest are not perturbed. FRAP, along with other optical fluorescence microscopy techniques, has been widely used to study and understand passive and active diffusion mechanisms through the NPC (Wei et al., 2003; Yang et al., 2004; Cardarelli and Gratton, 2010; Bizzarri et al., 2012).

In this work, we hypothesized that strain states in the NE cause changes in the structure of the NPC, thus in turn causing a significant change in the permeability of the NE to the traffic of transcription factors that are imported into the nucleus by passive diffusion. To quantify this effect, we set up a numerical model of the interaction between the NPC and the NE. We measured geometrical parameters of the NPC size/shape on reconstructed three-dimensional (3D) portions of the NE, in both roundish and spread cell configurations, by applying electron tomography (ET) analysis on cultured cells. We set up a computational model of the NPC-NE mechanical interaction in which the changes in NPC permeability due to the NE strains are caused by variations in the opening configuration of the nuclear basket, which in turn modifies the porosity of the NPC nuclear side in the NE.

To validate this model, we cultured cells in a substrate nanoengineered by two-photon laser polymerization which can maintain roundish cell nuclei due to the isotropic adhesion of cells to a 3D micro-lattice, called the “nichoid” (Raimondi, 2013). Cells adhering to the flat substrate surrounding the individual nichoids adhered in standard spread conditions to the flat 2D surface and showed spread nuclei. We transfected untagged GFP protein into MSCs grown in both roundish and spread conditions.

Our aim was to quantify, with FRAP experiments, how cell morphology affects the nuclear envelope permeability and hence the nucleocytoplasmic exchange of transcription factors. Finally, we compared the diffusion time constants predicted by the numerical model for roundish vs. spread cells with the constants measured by FRAP.

## Materials and methods

### Experimental protocols for NE reconstruction by scanning TEM (STEM)

#### Cell culture

MSCs were isolated from the bone marrow of adult rats (Zoja et al., 2012). Cells were isolated and cultured in alpha-MEM medium supplemented with 20% fetal bovine serum (FBS), 1% L-glutamine (2 mM), penicillin (10 units/ml), and streptomycin (10 μg/ml) at 37°C and in 5% CO_2_ (Euroclone, Italy). The culture medium was changed every 2–3 days and cells were used at stages 1–3 after thawing. The animal protocols used in this study comply with the institutional protocols for ethical use currently in force.

#### Sample preparation for STEM analysis

MSCs were plated (20,000 cells/cm^2^, *n* = 3) on glass coverslips (13 mm diameter) or 35 mm-Petri dishes. One day after plating, the culture medium was removed and cells were washed with phosphate buffered saline. To model the deformed (spread) configuration, MSCs were fixed for 2 h at room temperature with 1.5% glutaraldehyde in 0.1 M sodium cacodylate (pH 7.2), detached by scraping, centrifuged to recover the pellet, kept overnight at 4°C in 1.5% glutaraldehyde in 0.1 M sodium cacodylate and finally rinsed in 0.1 M sodium cacodylate (pH 7.2). To model the undeformed (roundish) configuration, MSCs were detached with trypsin, centrifuged to recover the pellet, fixed overnight with 1.5% glutaraldehyde in 0.1 M sodium cacodylate, and rinsed in 0.1 M sodium cacodylate.

#### STEM analysis

**Figure 2:**
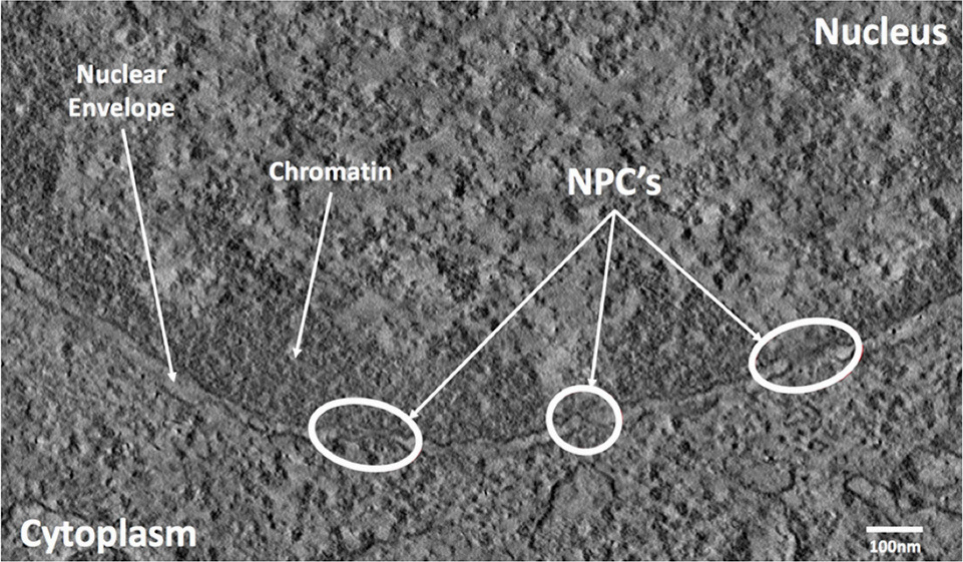
TEM image of the NE with NPCs (in circles).

After chemical fixation, MSCs cells in the spread and roundish configurations were washed several times in 0.1 M sodium cacodylate (pH 7.2), post-fixed in 1% osmium tetroxide in distilled water for 2 h and stained overnight at 4°C in an aqueous 0.5% uranyl acetate solution. After several washes in distilled water, the samples were dehydrated in a graded ethanol series, and embedded in EPON resin. Sections of about 70 nm were cut with a diamond knife (DIATOME) on a Leica EM UC6 ultramicrotome. Transmission electron microscopy (TEM) images were collected with an FEI Tecnai G2 F20 (FEI Company, The Netherlands). EM tomography was performed in scanning TEM (STEM) mode, using a high angular annular dark field (HAADF) detector on 400 nm thick sections of MSCs cells in both spread and roundish configurations. The tilt series were acquired from a ±60° tilt range. The resulting images had a pixel size of 1.85 nm as shown in Figure 2. The tomograms were computed with IMOD (version 4.8.40) (Kremer et al., 1996). Isosurface based segmentation and three-dimensional visualization on unbinned and unfiltered tomograms were performed using Amira (FEI Visualization Science Group, Bordeaux, France).

#### Nuclear envelope 3D reconstruction

**Figure 3:**
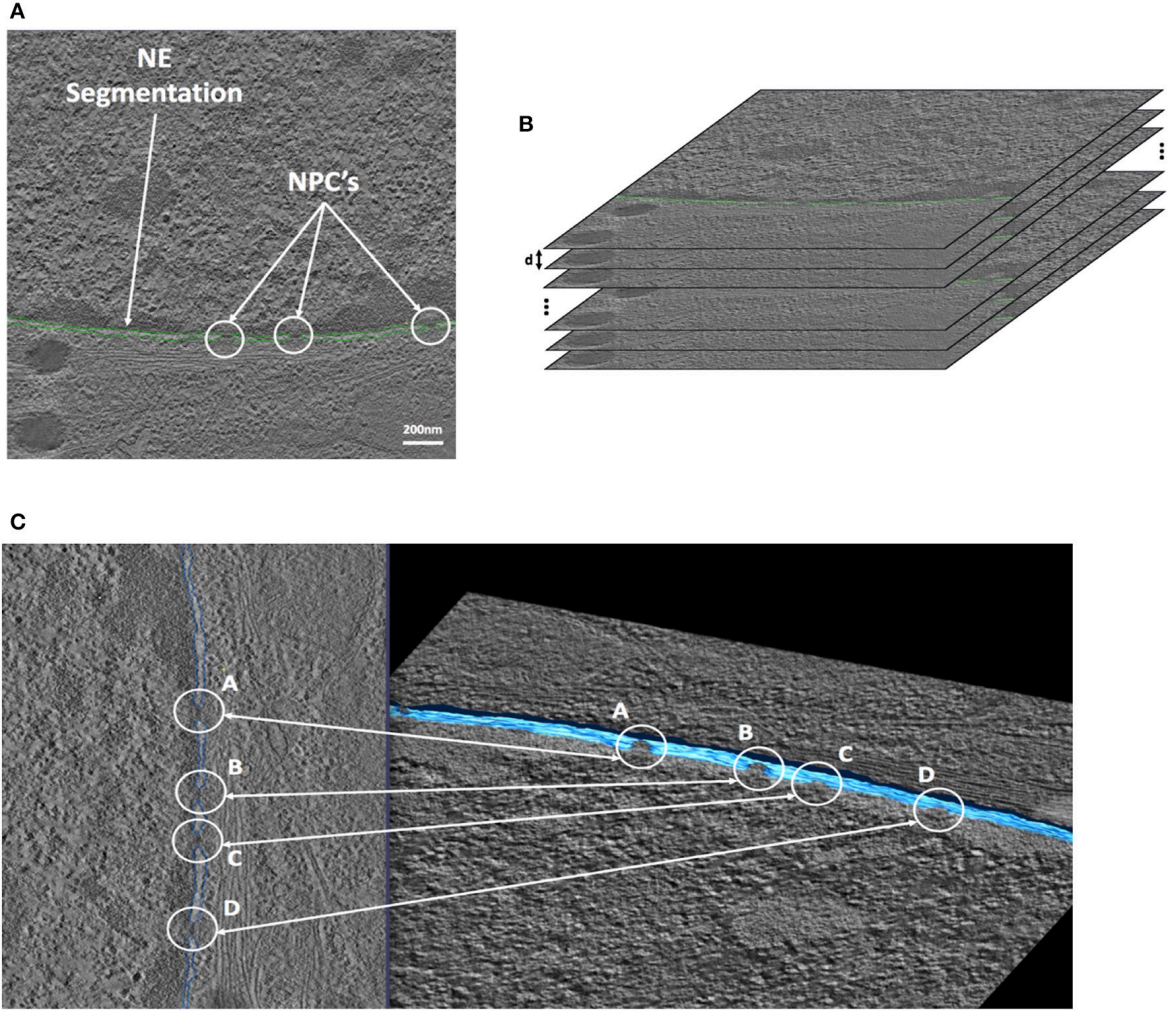
STEM Cell segmentation of the Nuclear Envelope and Pores. **(A)** Cell electron tomography with Nuclear Envelope segmentation (green). **(B)** Segmented cell tomographies for 3D reconstruction. **(C)** 1-slide segmentation of the NE (blue-left). 3D reconstruction (blue-right).

Open source image processing software, IMOD (Kremer et al., 1996), specialized in tomographic reconstruction developed by the University of Colorado was used to segment STEM images. Segmentation was performed manually on each slice. This process was guided by first locating the heterochromatin which is located very close to the membrane on the nuclear side (Figure 2). Figure 3A shows a typical slice segmentation detailing the location of several nuclear pores in the membrane. This process was followed for each slice as shown in Figure 3B. The nuclear envelope was then reconstructed by linear interpolation of the segmentation between consecutive slices (Figure 3C).

When the 3D reconstruction of the NE had been modeled, the geometrical data of the pores were measured directly using IMOD. Since the pore section is slightly elliptical, in order to obtain the area of each NPC, the two main diameters were obtained by measuring the pixel-by-pixel distances using IMOD. Additional post-processing regarding pore dimensions was performed in Matlab R2017b. Since we were measuring the main distances of the pixels between the mounted segmented slices, the main diameters were the closest approximations to the real diameters, due to the limited resolution of the STEM images. In order to obtain an accurate approximation of the pore area, a total of 16 and 19 pores were found in the reconstruction and measured for both spread and roundish configurations, respectively.

### Experimental protocols to analyse the diffusive process on cells

#### Cell culture on flat and 3D substrates

**Figure 4:**
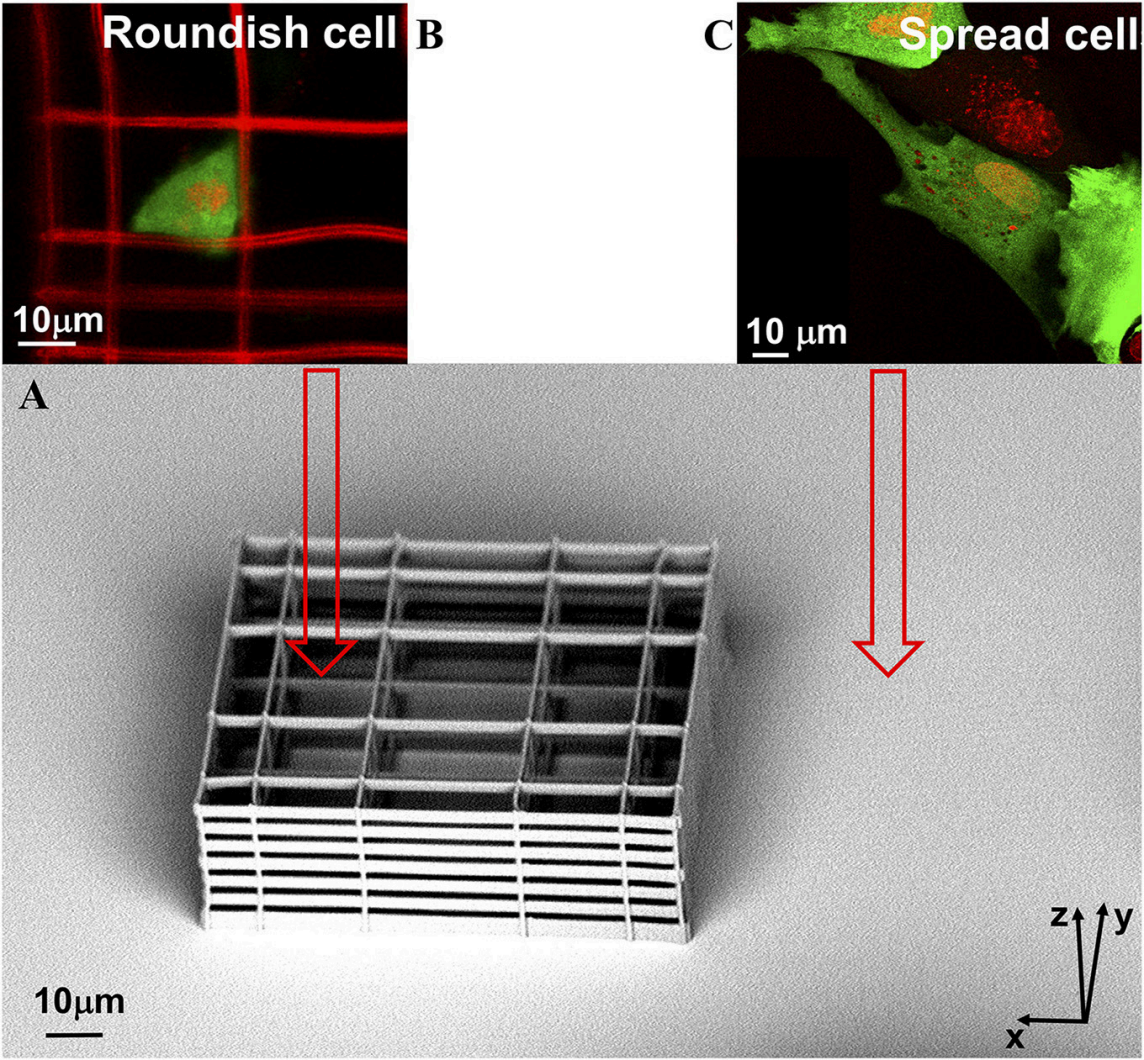
A representative sample. **(A)** one SEM image of the NICHE produced with the two-photon laser polymerization technique, and two images acquired in fluorescence confocal microscopy; **(B)** spread MSCs cells grown on the flat part of the sample; and **(C)** roundish MSCs cells grown in the NICHE. MSCs were seeded on the samples, transfected with GFP protein (green), and their nuclei were marked with the DRAQ5 dye (red). In the images of roundish cell, the niche is also visible—this is because the photoresist, SZ2080, is auto-fluorescent.

To recreate the two spread and roundish cell morphologies, cells were seeded on a chambered 160μ*m*-thick cover glass (Lab-Tek II, Thermo Scientific-Nunc) patterned with 3D “nichoid” structures fabricated using an organic-inorganic photoresist (SZ2080) by two-photon laser polymerization (Raimondi, 2013). In each chamber well, three niches were arranged in a triangular pattern, at a relative distance of 200 μ*m*. Individual niches were 30 μ*m* high and 90 × 90μ*m*^2^ in transverse dimensions. They consisted of a lattice with interconnected lines, comprising a complex structure with pores of a graded size (Figure 4). The lines had a uniform spacing of 15 μ*m* in the vertical direction, and a graded spacing of 10, 20, and 30 μ*m* in the transverse direction. Each niche was surrounded by four outer confinement walls, made up of horizontal rods spaced by 7.5 μ*m*, resulting in small gaps of 2 μ*m*, which allow the diffusion of nutrients, but prevent the cells from escaping outside the niche (Nava et al., 2015b).

Before cell seeding, samples were washed three times in deionized water, incubated overnight in ethanol 70%, washed three times in sterile deionized water and irradiated with UV light for at least 1 h. The samples were then treated with 0.01% of Poly-L-lysine solution (Sigma-Aldrich, Italy) to improve the cell adhesion, and again washed three times with sterile deionized water. Once dry, 20·10^3^ MSCs cells were seeded on each chamber. The day after, the cells were transient transfected with untagged GFP protein (pmaxGFP, Lonza, Switzerland).

#### Cell transfection

Cells were transiently transfected with GFP plasmid (pmaxGFP, Lonza, Switzerland) using the jet PRIME reagent (Polyplus, USA). A solution consisting of 0.5 μ*g* of DNA, 25 μ*l* of jet PRIME buffer and 1.12 μ*l* of jet PRIME reagent was prepared and kept at RT for 15 min. Cells were incubated with the transfection solution added to 400 μ*l* of antibiotic-free medium (alpha-MEM, 20% (FBS), 1% L-Glutamine; Euroclone, Italy). After 4 h, the solution was replaced with the complete medium. The day after, the medium was replaced with a DMEM phenol-red free medium (Lonza, Switzerland) containing 10% FBS, 1% Pen/Strep, 1% L-Glutamine. Nuclei were stained with 1 μ*M* DRAQ5 fluorescent probe (ThermoFisher, Italy) 10 min before the measurements.

#### Fluorescence recovery after photobleaching (FRAP)

FRAP measurements were performed with a confocal Laser Scanning microscope (Leica SP8, Germany) equipped with an Argon laser and a white laser, a 63X PlanApo oil-immersion objective (NA 1.4) and the incubator chamber. To identify the cell nucleus and choose the best plane to perform the FRAP measurement, DRAQ5 dye was detected using 8% of the Leica white-light laser (excitation 633 nm, emission 650–750 nm). For each cell, a region of interest identifying the section of the nucleus on which the FRAP measurement was later taken, was recorded to calculate the area. To acquire GFP protein emission, 0.2% of the 70% full power argon laser (excitation wavelength 488 nm, emission wavelength 500–580 nm) was used. Photobleaching of nuclear GFP was achieved by a single-point bleach (non-scanning) near the center of the nucleus with the 488 nm laser at full (100%) power. The time required to photobleach most of the nuclear fluorescence, without destroying too much of the cytosolic fluorescence, in flat cells was 3–5 s. In the case of cells grown in the niche, the maximum photobleaching time was 100 ms to avoid bleaching the GFP protein present in the cytoplasm.

**Figure 5:**
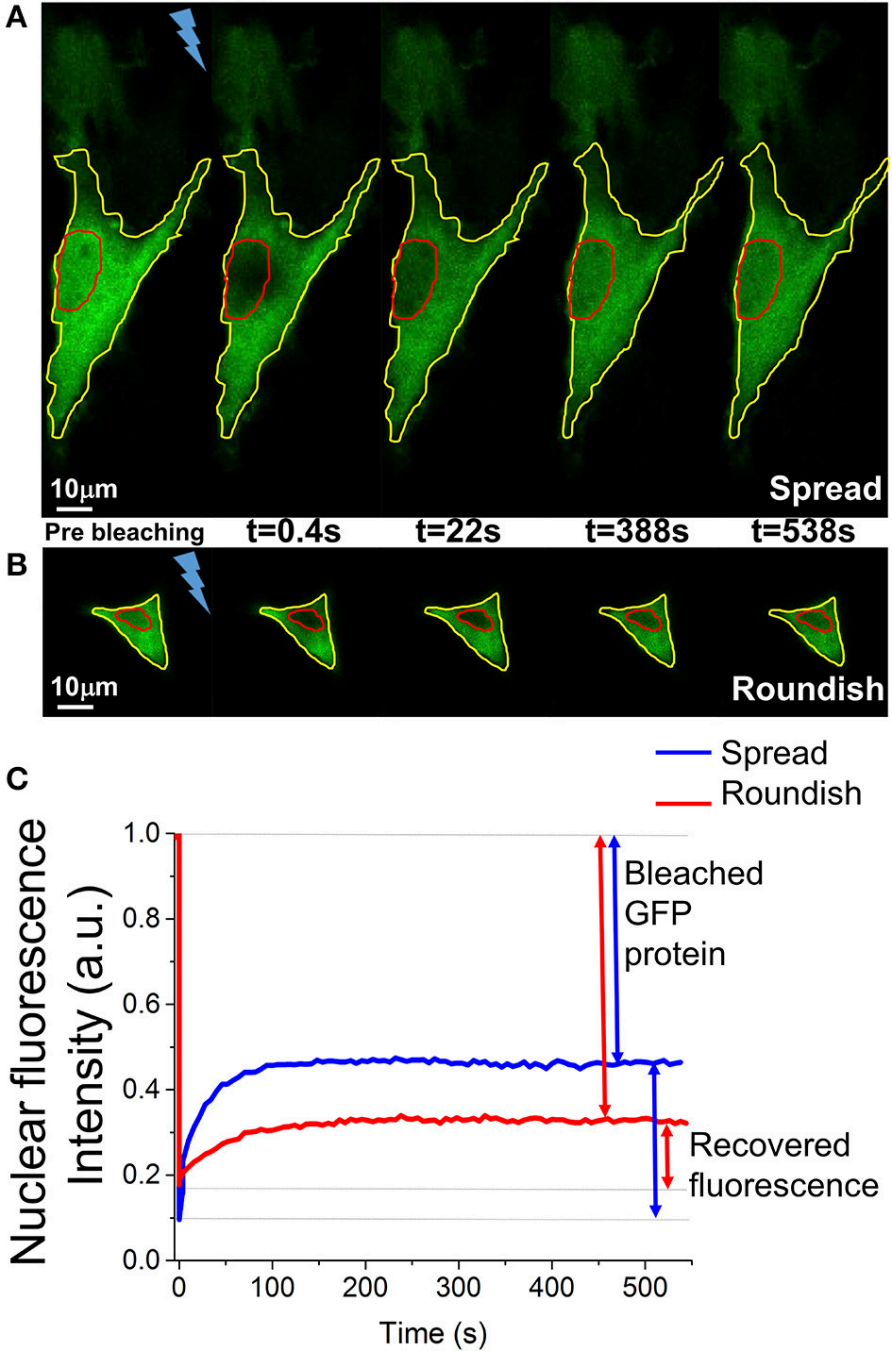
Example of a FRAP experiment. **(A,B)** Representative images of the nuclear fluorescence recovery in a spread and a roundish MSC. In red is highlighted the nucleus, in yellow the cell edge. The bolt represents the fluorescence bleaching performed with the high-power Argon laser at 488 nm. **(C)** Representative graphs of the FRAP curve in one spread cell (blue line) and in one roundish cell (red line). The GFP protein ratio bleached during the measurement and the ratio of fluorescent GFP protein recovered into the cell nucleus are highlighted.

Fluorescence recovery was measured starting a time-lapse acquisition within a few hundred milliseconds (382 ms) after the bleaching, acquiring 20 images every 191 ms and then 90 images every 6 s. Image size was 256 × 256 pixels and the scan speed was 700 Hz. Pinhole size was set to the value of 3.0 Airy, corresponding to a z resolution of 2.3 μ*m*. Ten acquisitions were performed for cells grown on a flat surface (spread cells) and cells grown in the 3D scaffold (roundish cells), respectively. The recovery of the fluorescence was evaluated for about 10 min, which is enough time to observe a fluorescence intensity plateau for a few minutes in the recovery curve. This plateau means that the exchange of dark and bright protein from the cytosol and the nucleus is indistinguishable. The curves associated with the image background was subtracted from each acquisition. To remove the intrinsic loss of fluorescence due to the imaging process, the nuclear fluorescence data were normalized to the total cell GFP-fluorescence intensity calculated with a ROI located on the cell edge (Figures 5A,B). Data were also normalized by the average value of the nuclear fluorescence intensity calculated over the last 30 s of the measurement. The curves obtained were then shifted to start at zero of the graph.

The fluorescence signal was assumed as being proportional to the GFP concentration and described by the function:

(1) $$\begin{array}{r} F\left(t\right)=F^{\infty }\left(t\right)+\left(F^{0}\left(t\right)-F^{\infty }\left(t\right)\;\right)e^{\frac{t}{t_{1}}} \end{array}$$

This average and normalized fluorescence recovery in the cell nucleus of the spread and roundish cells was calculated and was fitted (Origin Pro software) to a single exponential function using the following equation:

(2) $$\begin{array}{r} y=y_{0}+A_{1}e^{\frac{t}{t_{1}}} \end{array}$$

where *t*_1_ is the characteristic time (time constant) of the protein translocation from the cytosol to the nucleus, *A*_1_, is the difference between the nuclear fluorescence after the bleaching and the nuclear fluorescence at the end of the recovery, which corresponds to the fraction of protein involved, and *y*_0_ is the fluorescence background (see Table 2).

### Numerical modeling of the passive diffusion stretch dependency through the NE

Mechanical stretching of the nuclear lamina network (LN) plays a vital role in our hypothesis of stretch-dependent passive diffusion along the NE through the NPCs. This cytoplasmic fiber remodeling of the cytoskeleton (i.e., actin-myosin contraction) induces lamina deformation, therefore the NPC structure deforms at the nuclear ring (since it is directly linked to the lamina), and thus opening and closing the NR depending on the nuclear deformation. This effect causes an increase in flux in the case of the NR opening, since the effective area through the nuclear basket will become larger and thus leads to an increment on the velocity exchange of solutes. In addition, the flux of calcium released from the endoplasmic reticulum increasing through the NPCs, also increases the effective area of the distal ring (Stoffler et al., 1999a).

**Figure 6:**
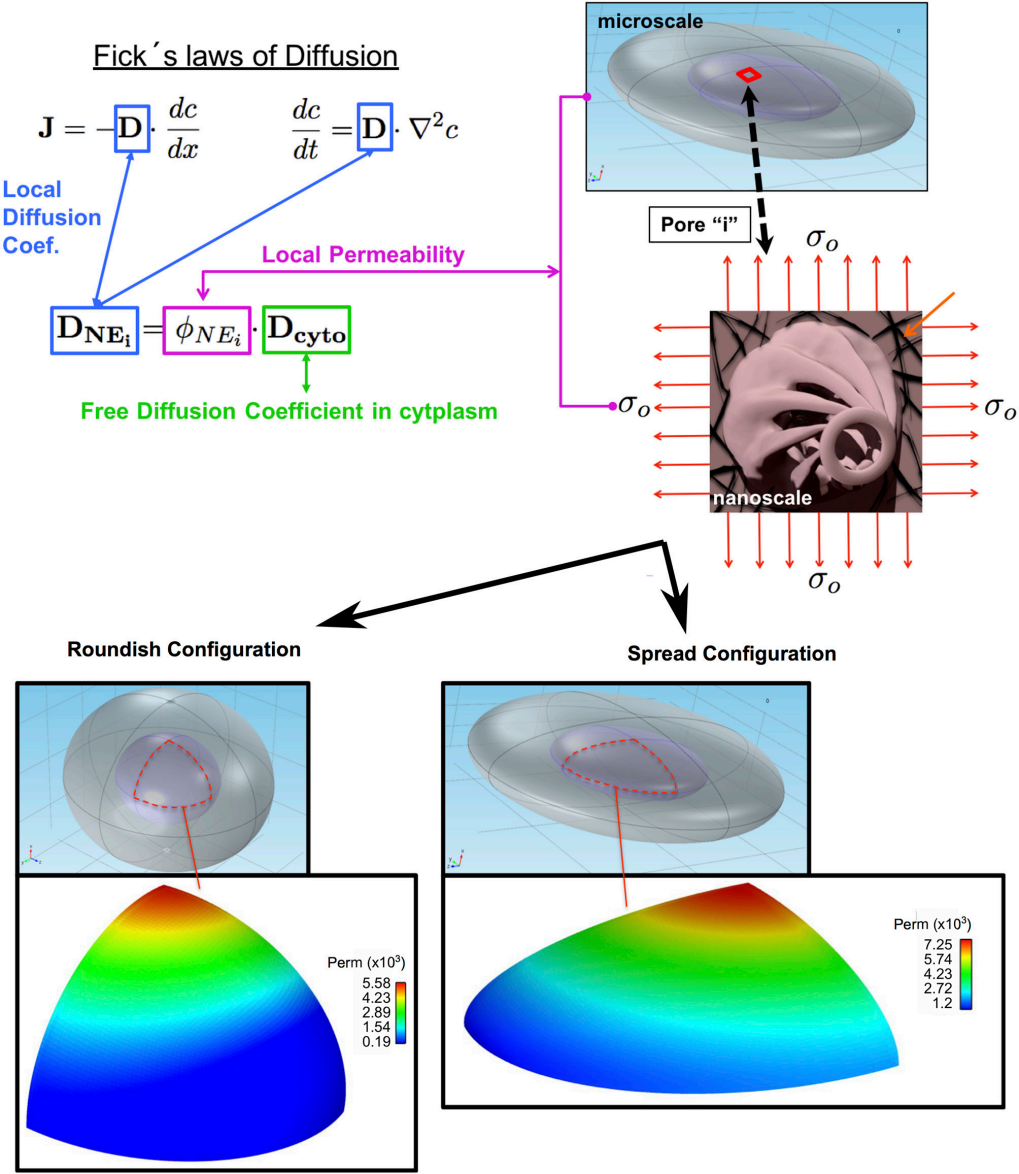
Stretch-dependent permeability model. Local permeability of the nuclear membrane varies according to the degree of deformation of the nuclear envelop separating the cytoplasm (gray) from the nucleus (light violet). The orange arrows in the biaxial stretching illustration shows the Lamina Network. The bottom panel depicts a typical nuclear envelope permeability distribution for cells with roundish and spread shapes.

It thus seems logical to suggest that the permeability of the NE increases due to the increase in the NPC's effective transport area (because of the mechanotransduction to the lamina network-NPC assembly) in the nuclear basket and the distal ring (DR). In this section, we propose a stretch-dependent model of the NE permeability, ϕ_*N*_*E*__*i*__. The model depends upon the local Green-Lagrange deformation tensor of the NE. Figure 6 summarizes the main aspects of the calculation of the local permeability, and thus the local diffusion coefficient.

The local diffusion coefficient *D*_*N*_*E*__*i*__ along the NE shown in Fick's Laws is calculated as a product of the Diffusion Coefficient of the GFP in the cytoplasm (assumed to be free diffusion) *D*_*cyto*_ and the local permeability ϕ_*N*_*E*__*i*__. To calculate the local permeability, as shown in Figure 6, we first calculated the local deformation at every point of the NE surface assuming that the nuclear envelope was isotropic and subject to a biaxial plane-stress distribution. We then used these values to calculate the effective transport area through the NPC by modifying the surface area of the basket. Finally, the local permeability is the ratio between the effective area of transport and the total area corresponding to a single NPC. The results predicted with this numerical model are compared with experiments described in section Confocal Analysis and Results of the GFP Transport Measurement.

Numerical simulations of the diffusion of GFP between the nucleus and cytoplasm were carried out in two different ellipsoidal configurations of the nucleus, roundish (cells proliferating in the niche) and flat (cells growing out in flat environment outside the niche), see Figure 4. The dimensions of the ellipsoidal main axis are taken from the experimental analysis previously reported by our group (Nava et al., 2015a).

#### Multiscale numerical model of stretch-dependent diffusion for the nucleocytoplasmic exchange of solutes

In order to determine the strain field in the NE, it is assumed that nucleus deformation occurs at a constant volume, as reported in (Nava et al., 2015a). In addition, it is assumed that the stress-free configuration of the nucleus corresponds to a sphere, whereas the deformed configuration is an ellipsoid. The mapping between the sphere and the ellipsoid surfaces can be written as

(3) $$\begin{array}{r} \begin{array}{c} x=\frac{a}{R}X,\\ y=\frac{b}{R}Y,\\ z=\frac{c}{R}Z, \end{array} \end{array}$$

where *R* is the radius of the reference sphere, *a, b, and c*, are the semi-axes of the deformed ellipsoid, *X, Y, Z* are the coordinates of the points in the nucleus in the reference sphere, and *x, y, z* are the coordinates of the points in the nucleus in the deformed configuration. This mapping can be parametrized in terms of spherical coordinates θ (polar angle) and φ (azimuthal angle)

(4) $$\begin{array}{c} x=a\cos\theta \sin\varphi ,\;X=R\cos\theta \sin\varphi ,\\ y=b\sin\theta \sin\varphi ,\;Y=R\sin\theta \sin\varphi ,\\ z=c\cos\varphi ,\;Z=R\cos\varphi . \end{array}$$

As already noted, the parametrization in Equation (4) provides a one-to-one mapping between the reference and deformed configuration.

The in-plane deformation of the nuclear envelope between the reference sphere and the deformed ellipsoid can be calculated using standard continuum mechanics theory from the exact mapping described in Equations (3) and (4). In this regard, the principal in-plane Green-Lagrange deformations are given as

(5) $$\begin{array}{c} E_{1}=t_{\theta }\cdot \left(E\cdot t_{\theta }\right),\\ E_{2}=t_{\varphi }\cdot \left(E\cdot t_{\varphi }\right), \end{array}$$

where **t**_θ_ and **t**_φ_ are tangent vectors along the polar and azimuthal direction in the reference sphere, respectively

(6) $$t_{\theta }=\left(\begin{array}{c} -\sin\theta \\ \cos\theta \\ 0 \end{array}\right),\;t_{\varphi }=\left(\begin{array}{c} \cos\theta \cos\varphi \\ \sin\theta \cos\varphi \\ -\sin\varphi \end{array}\right),$$

and **E** is the Green-Lagrange deformation tensor

(7) $$E=\frac{1}{2}\left(F^{t}F-I\right),$$

with $\text{F}=\frac{\partial \text{x}}{\partial \text{X}\;}$ the deformation gradient obtained from the mapping in Equation (3). Substituting in Equation (5) results in the following expression for the principal in-plane Green-Lagrange deformations

(8) $$\begin{array}{c} E_{1}=\left(\frac{a^{2}}{R^{2}}-1\right)\cos^{\text{2}}\theta \cos^{2}\varphi +\left(\frac{b^{2}}{R^{2}}-1\right)\sin^{\text{2}}\theta \cos^{2}\varphi \\ +\left(\frac{b^{2}}{R^{2}}-1\right)\sin^{2}\varphi ,\\ E_{2}=\left(\frac{a^{2}}{R^{2}}-1\right)\sin^{2}\theta +\left(\frac{b^{2}}{R^{2}}-1\right)\cos^{2}\theta . \end{array}$$

**Figure 7:**
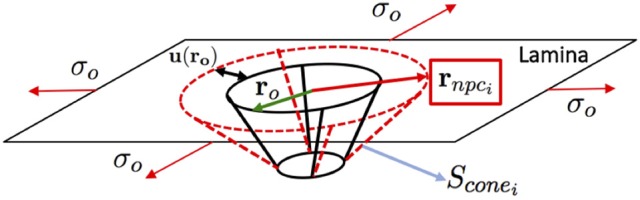
Lamina Network-Nuclear Ring-Nuclear Basket-Distal Ring (LN-NR-NB-DR assembly) illustration of the equibiaxial stress configuration of a single NPC.

Figure 7 shows the “Lamina-NR-basket-DR” assembly considered in the model. Since the radius of curvature of the NE is larger than the nuclear pore dimensions (radius of a curvature ratio of 100:1), the pore in the nuclear lamina can be modeled as a plate with a circular hole under biaxial stress which allows for an analytic solution (Mal and Singh, 1991). We also assume that the NE deformation induces an equibiaxial state of stress/deformation on every pore in which the stress is proportional to the trace of the in-plane Green-Lagrange deformation i.e., *tr*(**E**_*i*_) = *E*_1_+*E*_2_. Note that, in the case of plane-stress, the trace of the in-plane Green-Lagrange tensor in small deformations is proportional to the relative area change. Following the solution in Mal and Singh (1991), the change in the nuclear ring radius (see Figure 7), *r*, is given by

(9) $$\Delta r=2\frac{tr(E_{i})}{\left(1-\nu \right)}r_{0},$$

where *tr*(**E**_*i*_) is the trace of the local in-plane Green-Lagrange deformation tensor, ν is the Poisson ratio of the lamina, assumed as 0.3, and r_0_ is the undeformed NR radius. Hence, the radius of the deformed NR after deformation is

(10) $$r_{npc}{}_{i}=r_{0}+\Delta r=r_{0}\left(1+2\frac{tr(E_{i})}{\left(1-\nu \right)}\right).$$

With these calculated radii of the NR in the deformed configurations, it is possible to obtain the lateral surface of the nuclear basket, *S*_*con*_*e*__*i*__, (see Figure 7) and thus the effective area of the transport of solutes through one single pore as

(11) $$A_{npc_{i}}\left(E_{i}\right)=A_{DR}+\left[S_{cone_{i}}\left(E_{i}\right)-\left(1-A_{e}\right)S_{cone_{0}}\right],$$

where *A*_*DR*_ is the area of the Distal Ring, *S*_*con*_*e*__0__ is the value of the lateral surface of the nuclear basket in the undeformed configuration, and *A*_*e*_ is a surface correction factor accounting for the pillars connecting the NR and DR which reduce the effective area of transport. In the model, *A*_*e*_ is set to 0 which implies that the lateral surface of the basket is closed in the undeformed configuration. Once the effective transport surface area has been computed, the local permeability and the local Diffusion Coefficient can be readily calculated as:

(12) $$\phi_{NE_{i}}=\;\frac{A_{npc_{i}}\left(E_{i}\right)}{A_{i}}=\;\frac{A_{npc_{i}}\left(E_{i}\right)}{A_{NE}}N_{P}=\frac{A_{npc_{i}}\left(E_{i}\right)}{A_{NE}}\rho_{npc}A_{NE_{0}}.$$

where $A_{i}=\frac{A_{NE}}{N_{P}}$ is the area ratio corresponding to a single NPC, *A*_*NE*_ is the total area of the nuclear envelope, *N*_*P*_ is the total number of pores, ρ_*npc*_ = *N*_*P*_/*A*_*N*_*E*__0__ is the pore density (number of pores per unit area), and *A*_*N*_*E*__0__ is the zero-stress (spherical) surface area of the NE. With the expression of the permeability in Equation (12), the effective nuclear membrane diffusion coefficient can be calculated as:

(13) $$D_{NE_{i}}=R\phi_{NE_{i}}D_{cyto},$$

where *D*_*cyto*_ is the GFP-FRAP free diffusion coefficient and *R* is a “traffic resistance” parameter that takes into account the resistance to the trafficking of high molecular weight cargos through the pore (pores are always full of molecules passing through them). Therefore, the final permeability is reduced due to the resistance. Note, however, that *R* is considered to be the same for the roundish and spread configurations. In our case, we found that 96.1% flux resistance was optimal to fit the numerical model to the experimental results.

The presented model is used to compute local values of diffusion *D*_*N*_*E*__*i*__ to be included in a finite element model of the passive nuclear transport (see Figure 6, bottom panel). As can be seen, the finite element models of diffusion consist in a symmetric octant of a solid ellipsoidal stem cell (created using Comsol Multiphysics), one for a roundish and another for spread configurations. Such models were meshed with a total of 2,91,420 hexahedral elements and 3,02,236 nodes for the roundish cell, and 4,62,264 elements with 4,77,468 nodes for the spread cell. We divided the FE models in three main parts: (i) an external thin layer of elements that represents the nuclear envelope, in which the different calculated values of *D*_*N*_*E*__*i*__ were added in each of the elements (accounting for the permeability of the NE-NPC). (ii) The full nucleus and cytoplasm volumes in which free diffusion was considered. The model simulates (run in Abaqus 6.14-1) the transport of GFP from the cytoplasm to nucleus through the NE until equilibrium is reached. (iii) Finally, a post-process of the simulation results is performed suing Python-Matlab to calculate the total concentration in the nucleus vs. time.

Since the numerical finite element model is meant to be able to fit the experimental results, the different parameters in the model were selected to be of the same order of magnitude as reported in the literature (Stoffler et al., 1999b; Beck et al., 2004; Moussavi-Baygi et al., 2011; Maimon et al., 2012; Adams and Wente, 2013; Bui et al., 2013; Eibauer et al., 2015). In particular, the SR radius was taken as 0.01 μ*m*, the initial NR radius was 0.04 μ*m*, the DR radius as 0.0 μ*m* (since the DR is assumed not to be influenced by mechanical deformations of the NE), and a basket length of 0.075 μ*m*. In addition, the value of GFP-FRAP free diffusion coefficient was taken as *D*_*cyto*_ = $31\;\frac{m^{2}}{s}$ (Baum et al., 2014), and the nuclear pore density $\rho_{npc}=10\;\frac{pores}{{\mu m}^{2}}$ (Bizzarri et al., 2012), with which we obtain a total of 2908 NPCs/nucleus.

## Results

### Nuclear envelope 3D reconstruction

**Figure 8:**
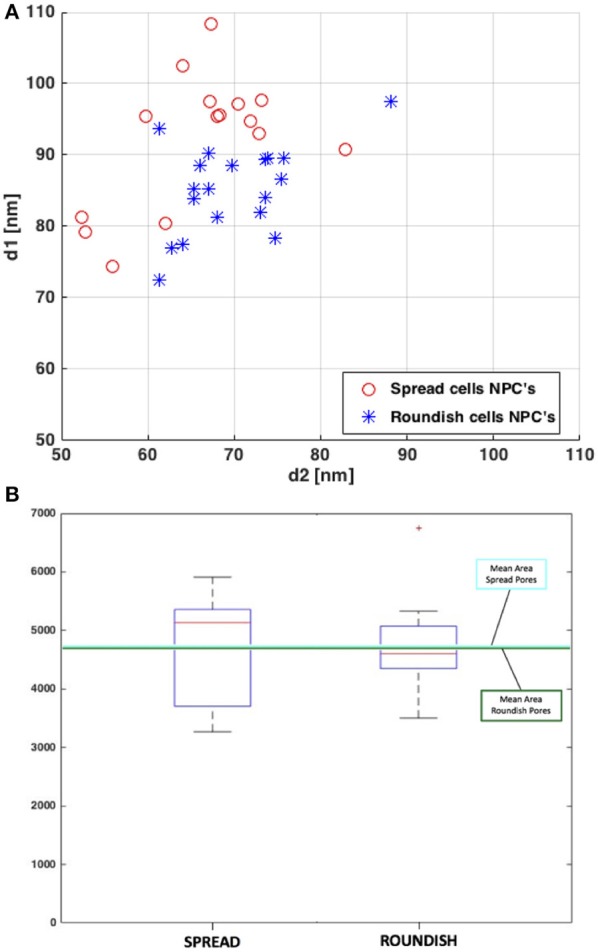
**(A)** Elliptical principal measured diameters of the SR of the NPCs. **(B)** Box plot of the resulting areas of the NPCs. The average area values for both cell configurations are plotted in light blue (for spread cells) and dark green (for roundish cells).

Table 1 shows the pore diameters and areas obtained from the 3D reconstruction. It is worth mentioning that in line with the STEM, the diameters measured correspond to the distance at the SR level since it is only possible to visualize the NE rather than the NPC itself. The mean diameter values show a higher deformation of the pore area in the spread cells compared to the roundish cells. These differences in diameter between the spread and roundish configurations are shown in Figure 8A. Despite this change in diameter, both the spread and roundish configurations show similar pore areas values, see Table 1, with a higher dispersion of values in the spread cells as shown in Figure 8B. The difference in the pore area between the roundish and spread configurations was tested with a paired, two-sided signed rank test that founded no statistical differences (*p* = 0.19). These results reinforce the strong hypothesis that the effective area of diffusive transport relies on the NR-basket-DR assembly due to the deformation of the NE-Lamina Network (directly linked to the NR). Thus, the SR and the transmembrane ring become the main substructures on which the main stiffness of the whole NPC depends.

**Table 1:** Diameters of the NPCs of both roundish and spread configurations.

	**Spread**			**Roundish**		
	**d1 (nm)**	**d2 (nm)**	**NPC area**	**d1 (nm)**	**d2 (nm)**	**NPC area**
Mean	92.17	64.83	4723.40	85.31	69.78	4690.60
Std	9.07	9.12	944.33	6.21	6.60	696.93

*Data are reported as mean and standard deviations*.

### Confocal analysis and results of the GFP transport measurement

One day after MSC transient transfection with GFP protein, FRAP experiments on cell nuclei were performed. GFP-transfected cell images are reported in Figures 4B,C which shows images acquired before the FRAP experiment of a cell grown in the niche, and of a spread cell adhered to the glass substrate shown in Figure 4A. The pictures show that the cell morphology drastically changes depending on the environment, flat glass substrate-−2D— or the NICHEs-−3D—, in which the cell is grown.

FRAP experiments were performed as reported in the Materials and Methods (see section entitled FRAP) and representative images of the nuclear fluorescence recovery are shown in Figures 5A,B. The graph in Figure 5C shows the relative curves of the fluorescence recovery in the cell nucleus. Each of these functions shows the initial value of nuclear fluorescence, on which the curves were normalized, the bleaching time and the recovery of the nuclear fluorescence over time. As shown in the graph, despite the GFP being a non-interacting protein with other cellular components, the recovery of the fluorescence does not reach the initial intensity because, during the bleaching time, many GFP-proteins (in and outside the nucleus) are irreversibly bleached. In particular, in the 3D cell configuration, it is not possible to reach very low level (80% of bleaching) of fluorescence in the nucleus without destroying the cellular fluorescence. The bleaching time needs to be reduced from a few seconds (for the spread cells) to 100 ms and the total recovery is calculated considering only 30% of the initial fluorescence.

**Figure 9:**
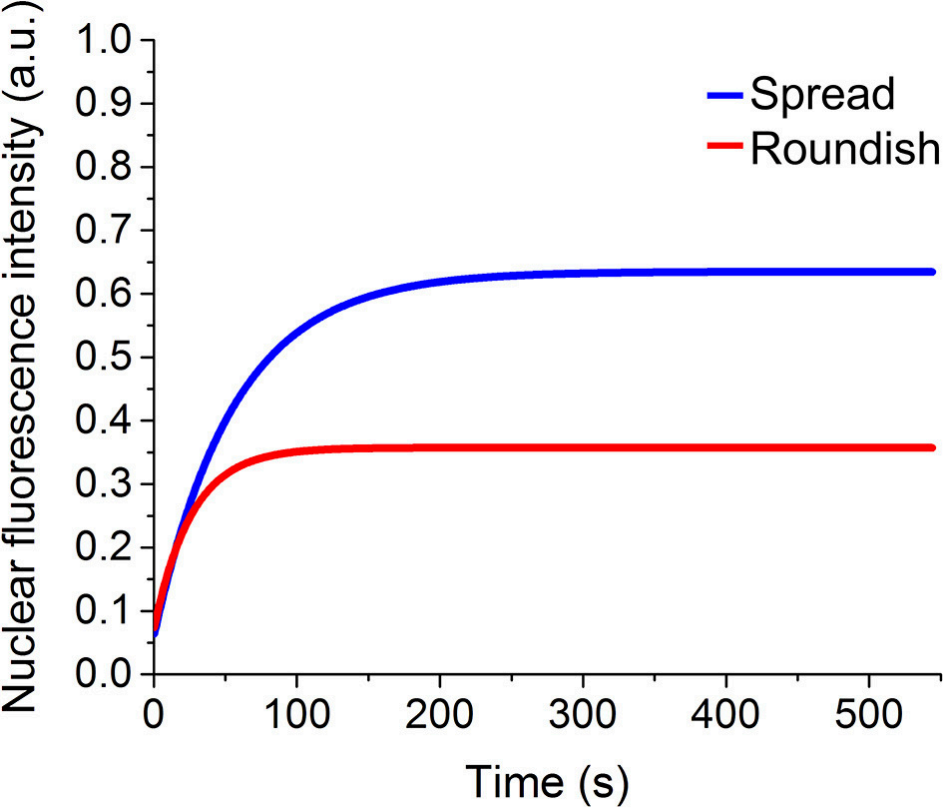
Fit of the experimental recovery of GPF protein in MSCs grown in different conformations.

Figure 9 shows the fit of the recovery curves of the spread and roundish cells. The bleached area in the two populations is on average $A_{spread}=123\;\pm \;34\;\mu m^{2}$, $A_{Roundish}=40\;\pm \;13\;\mu m^{2}$. The curves are well fitted with a monoexponential function, as demonstrated by the statistical analysis (reduced-χ^2^ spread cells = 0.979 reduced-χ^2^ roundish cells = 0.946). The parameters extracted from the fits are reported in Table 2, which highlights the characteristic diffusion time of the GFP translocation between the cytosol and cell nucleus of the spread and roundish cells (*t*_*spread*_ = 56 ± 2.6 *s*, and *t*_*roundish*_ = 26 ± 2.5 *s*).

**Table 2:** Parameters of the mono-exponential function used to fit the fluorescence recovery curve on spread and roundish cells: *t*_1_ is the characteristic time of the GFP protein translocation from cytosol to the nucleus; *A*_1_ corresponds to the fraction of protein involved in the exchange; and *y*_0_ is the fluorescence background.

**Sample**	**y_0_ (a.u)**	**A_1_ (a.u)**	***t_1_* (s)**	**σ_y_0__(*a*.*u*)**	**σ_A_1__**	**σ_t_1__ (s)**
Roundish cells	0.6	−0.6	26	0.004	0.008	2.6
Spread cells	0.4	−0.3	56	0.003	0.007	2.5

### Numerical simulations of stretch dependent diffusion of GFP

**Figure 10:**
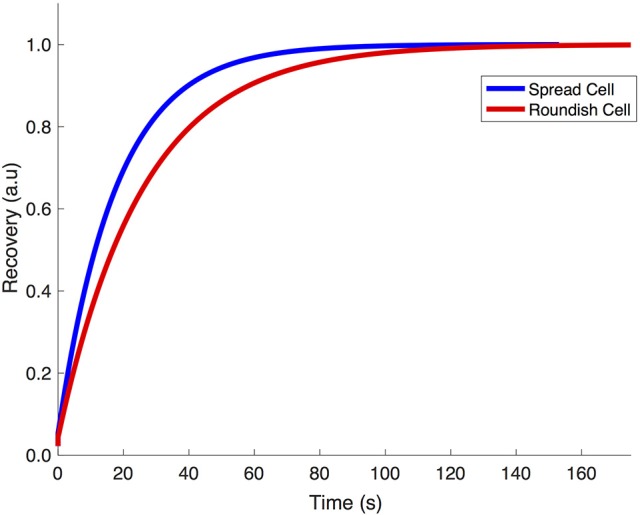
Computational simulation of the recovery of GFP transcription factor in spread vs. roundish configuration.

Figure 10 shows the finite element simulation results of the recovery of GFP by the stretch-dependent diffusion model previously described for both spread (blue) and roundish (red) configurations of the nucleus. The faster recovery of the spread compared to the roundish nucleus is clear, since the level of deformation in the NE of the spread nucleus is larger than in the roundish nucleus, and therefore more permeable (see Figure 6 bottom panel). According to the results in Figure 10, the corresponding characteristic time for both spread and roundish configurations were found to be *t*_1*spread*_ = 17.2 *s* and *t*_1*roundish*_ = 25.4 *s* which were very similar to those obtained experimentally. These results were obtained using the aforementioned structural/dimensional values of the pores and the corresponding permeability. The difference in recovery times is only due to the degree of modulation that the deformation of the NE exerts on the NE permeability.

## Discussion

To the best of our knowledge, there are no papers in the literature that specifically use computational mechanics and numerical analyses to demonstrate strain-dependent passive diffusion through the NE. Instead the focus has been on the mechanisms that lead with the active transport of cargoes through the NPCs, see for example (Moussavi-Baygi et al., 2011; Azimi and Mofrad, 2013; Mahboobi et al., 2015). The work by Nava et al. (2015b) treated the passive diffusion of solutes between the nucleus and the cytoplasm as strain-dependent. In their analysis, the whole nucleus is deformed and assumed as a permeable material. In our literature search we found no other studies on this topic.

The multiscale numerical model presented in this paper, is thus the first attempt to directly analyse the passive diffusion of small molecules through the deformed NPCs (nano-level) at the nuclear envelope scale (micro-level) by including a strain-dependent variable permeability barrier in the NE. Our results highlight the potential of our numerical model to describe the passive transport through the nuclear membrane, that is, the passive diffusive flux of small molecular weight particles. Note that, since the DR diameter is a variable parameter of the numerical model, it may also account for calcium effects on distal ring opening. However, in our numerical simulations, we set the DR diameter to 0 μ*m* in order to only analyze the passive diffusion mechanical dependency. This is because the DR is chemically opened/activated by a calcium flux through the NPC (Stoffler et al., 1999a; Wang and Clapham, 1999).

An important limitation regarding our model is the use of small deformation theory for the NPC and isotropic linear elastic behavior for the NE-lamina network. We assumed such mechanical properties due to the lack of available data for the NE-lamina-NPC assembly. We also consider this numerical model as a first attempt to demonstrate our hypothesis, and we believe that more complex material properties should not greatly qualitatively modify our final results. However, these assumptions require further research in order to obtaining more accurate results that would reinforce our final hypothesis.

Fluorescence recovery after photobleaching belongs to a class of measures based on photoperturbation. This means that only the optical properties of the protein of interest are changed and, after the perturbation, the protein redistribution in space is monitored in time-lapse. This class of measures is also known as ensemble-averaging, in fact it is possible to obtain results over a relatively long time (from hundreds of milliseconds to minutes) and they are the result on average of the behavior of many molecules. This means that the measure masks the fast diffusion process or hides the properties of sub-populations. In general, their results need to be coupled with a mathematical model to help in the data interpretation.

Usually, in FRAP experiments, a high concentration of the protein of interest is expressed in a live cell fused with a fluorescent protein (GFP protein for example). A small area, in a single cell compartment, i.e., the region of interest, is permanently bleached by a strong laser illumination, and the redistribution of the fluorescence in the entire cell is monitored by low intense excitation (as in Figures 5A,B). If the protein of interest is immobile, the bleached area will remain dark. On the other hand, if it is mobile, then a redistribution between the fluorescent and bleached protein happens between the region of interest and the rest of the cell.

In order to study the mobility of a nuclear protein, due to the confocal\ wide field microscopy set up, the bleaching takes place in a cylindrical volume of a few microns along the z axis, which include the cell nucleus and also the cytoplasm. This involves the destruction of the fluorescence of a small portion of the protein in the cell cytoplasm. However, this does not affect the measure because in cells grown on a 2D flat surface (like our spread cells), these cytosolic bleached volumes were very small, because the nucleus generally fills the space between the upper and lower plasma membranes.

This technique has been used to evaluate the protein redistribution between two different cell compartments i.e., between the cell cytoplasm and nucleus. In this experimental configuration, as in our experiments, a wide as possible photobleached area within the nucleus was used, to ensure that the entire nucleus was photobleached, and the nuclear intensity recovery, as a consequence of the protein transport between the cytoplasm and the nucleus, was monitored. In this case, the prolonged GFP fluorescence recovery of the nuclear compartment (tens of seconds), compared to the GFP free diffusion in the nucleus or in the cell cytoplasm (2 s) (Lippincott-Schwartz et al., 2001; Wei et al., 2003; Sprague and McNally, 2005; Bizzarri et al., 2012), is due to the restricted diffusion across the nuclear envelope, which is in line with the diffusion through the open NPCs (~0.01 of total NE surface area, Wei et al., 2003). This is also shown in our work, from the graphs in Figure 5C.

Our results also show that our experimental conditions—the long bleaching time performed on spread cells, and the large volume of cytoplasm above and below the nucleus in the roundish cells—induced a high ratio of fluorescence protein disruption. This is also supported by the fact that we are working with a single GFP which is a non-toxic inert protein that does not interact with nuclear and cytosolic components, and therefore which does not show an immobile fraction during the FRAP measures. As is evident from our results on the GFP translocation, the characteristic time between the cytoplasm and the nucleus of the spread cells is comparable with those of the literature performed on cells grown on a standard flat substrate such as a glass coverslip (Wei et al., 2003; Sunn et al., 2005; Bizzarri et al., 2012). At the same time, we were unable to compare the results obtained on roundish cells because in the literature there are no similar experiments performed on cells grown on 3D scaffolds.

A comparison of the characteristic recovery time of the nuclear fluorescence of these two cell populations, led to the unexpected result: the fluorescence recovery was faster for the round cells than the spread cells. A careful evaluation revealed that we were evaluating the fluorescence recovery on a single (3 μ*m* in thickness) plane of the cell, in which the area of the nucleus differed greatly between spread cells and roundish cells ($\frac{A_{spread}}{A_{roundish}}\;=\;3$). This means that a larger number of particles have to translocate and therefore it takes a longer time for the GFP to diffuse over the area of the spread cell nucleus. However, an experimental analysis performed on MSCs cells grown on a glass flat substrate and in the nichoid did not show a significant difference in the nuclear volume (Nava et al., 2015a), which suggests that the number of proteins that translocate from the cytosol to the cell nucleus in FRAP experiments does not strongly influence the measure. Other factors therefore need to be considered that may affect the recovery time, such as a strong modulation of the number of pores, or a reduction/increment in the effective nuclear surfaces.

None of the results presented in this manuscript i.e., the NPC spoke ring area via STEM analysis, the numerical parametric finite element diffusion model and FRAP experiments with the confocal microscopy, contradict the hypothesis that the deformation/strain of the nuclear envelope induces structural modifications in the NPCs and thus directly affects the passive diffusion of molecules. These results can be directly linked to the existence of small diameter secondary channels through the NPC that may allow small molecules such as ions to pass from the cytosol to the nucleus. In the case of a full blockage of the NPC due to high trafficking and deformation, it therefore allows the ions to open the distal ring and thus, to increase the flux through the NPC. We already mentioned this in a previous paper (Garcia et al., 2016) and referenced the works of (Maimon et al., 2012; Eibauer et al., 2015) which showed such secondary channels.

A major limitation of the work discussed in the present paper is that we were forced to use two different techniques to maintain roundish cells in the experiments. The cells were fixed in suspension to keep them roundish for the STEM reconstructions used to estimate the NPC dimensions, and they were cultured in the nichoid substrate for the FRAP measurement of the GFP nuclear import. In fact, the nichoid substrate is made of a fragile polymer that cannot be sectioned for STEM preparation without it being destroyed. Moreover, cells cannot be measured by FRAP for nuclear import flows while in suspension.

Reducing the cell adhesion sites by limiting the area of the adhesion substrate available for integrin binding, which is a similar approach to suspension culture, is a widely-accepted method used to induce a roundish cell morphology in mechanobiological studies (Badique et al., 2013). However, reducing the adhesion sites to maintain cells in a roundish morphology is likely to down-regulate the activation of mechanobiological transcription factors and other signaling molecules linked to the pathways activated by focal adhesions. Thus, inducing cell adhesion to a 3D scaffold is preferable to limiting the cell adhesion sites, for mechanobiology investigations. However, here we did not measure the activation or nuclear imports of transcription factors or signaling molecules, we only measured the nuclear imports of the GFP protein, expressed in the cell following transfection regardless of the cell morphology. In designing the experiments, we basically assumed that nuclear pore activation was primarily affected by NE local strains induced by nuclear deformation, regardless of the means used by the cell to adhere to its environment.

Another important limitation of our study is that in the nichoid culture model, the mechanical properties of the adhesion substrate were different for the spread and roundish cells. Spread cells adhered to glass, with a Young's modulus of around 80 GPa, while the photo-polymerized nichoid micro-lattice has a Young's modulus in the order of 0.138 GPa, i.e., three orders of magnitude less stiff than glass. The stiffness of a substrate to which the cell adheres is known to correlate significantly with the fate of several stem cell types, including MSCs, thanks to pioneering demonstrations by the research groups of Discher and Engler. It could thus be argued that the differences between the roundish and spread cells that we measured by FRAP in terms of nuclear flows are related to differences in the adhesion substrate stiffness. However, our previous findings using the nichoid cell culture model (Nava et al., 2015b) suggest, in addition to the stiffness theory, that the substrate stiffness combines with the substrate architecture in generating an adhesion configuration for the cell, which can be either isotropic (roundish) or very far from isotropic (spread), which correlates with the shape of the cell's nucleus.

We deduced that the level of nuclear isotropy induced by the combination of stiffness and architecture of the adhesion substrate, and not the substrate stiffness itself, was indeed the primary parameter correlating with the cell fate. In order to move from correlation to causation, in this work we introduced the hypothesis that, when the cell spreads, a primary mechanism activating the master switch between cell programs is the NPC stretch activation leading to a sudden increase in the permeability of the NE to purely diffusive signaling molecules. Our modeling approach, far from negating the primary role of substrate stiffness on cell fate, integrates substrate stiffness with its 3D architecture, thus providing a mechanistic interpretation of this effect, which is well corroborated by *in vivo* measurements of changes in diffusive nuclear flows due to nuclear morphology.

In future works, we will test our hypothesis on the key transcription factors involved in MSC differentiation. Many of these are molecules are in the range 40–70 kDa, which can diffuse freely (without consuming chemical energy) through NPCs. For example, the molecular weight of MyoD, a key myogenic transcription factor, is in the range 34–45 kDa. The molecular weight of Cbfa1 (also called Runx2), a transcriptional activator of osteoblast differentiation, is 55 kDa. Thus, these key transcription factors may diffuse freely through the NPCs. Thanks to the mechanobiology model developed here, we will be able to computationally predict their nuclear import flows on the basis of their molecular weight, and we will be able to interpret and validate these predictions with FRAP measurements on cells cultured in the nichoid model. To perform FRAP measurements, we will fuse the transcription factors with an inert fluorescent protein, such as GFP which is only 27 kDa in size, enabling us to still fall within the 70 kDa limit in the overall molecular weight of the fused protein, for NPC translocation based on passive diffusion. In fact, we selected GFP in this work because of its very limited size, as it falls well below the 40 kDa lower limit known for the passive diffusion of molecules through NPCs.

We also plan to characterize, in cells of a different morphology, the activation of gene expression induced by the nuclear translocation of the transcription factors of interest. However, a quantitative correlation between NE permeability and the up-regulation of gene expression is not expected, because up-regulation very much depends on the degree of chromatin packing influencing DNA accessibility to the chemical binding of the transcription factors.

In conclusion, here we have proposed a fundamental mechanism which uses nuclear mechanics to orchestrate the response of progenitor cells to the architectural properties of the extracellular environment. Our data show that cell stretching modulates the characteristic time needed for the nuclear import of a small inert molecule, GFP. What still needs to be proven is whether this modulation effect is due to an opening of the distal ring. We also still need to prove that a transcription factor with a comparable size to GFP would be subjected to the modulation effect that we found for GFP.

If further verified on specific transcription factors involved in MSC differentiation, this idea could thereby contribute directly to the definition of better differentiation protocols for MSCs, primarily based on guiding the spontaneous tendency of stem cells to differentiate in culture, by the mechanical cues provided by “physically” biomimetic culture niches. A new research field that could be impacted by our hypothesis is the fate control of induced pluripotency stem (iPS) cells. The iPS technology consists in converting adult somatic cells, usually fibroblasts or epithelial cells, to a pluripotent phenotype using genetic engineering. Despite the high potential of iPS to revolutionize medicine, to date there are very few successful re-differentiation protocols regarding mature phenotypes for these cells. Neurobiology is the only field where there are stable differentiation protocols. Our hypothesis could produce the knowledge and technology, the nichoid culture substrate, to direct the differentiation of iPS to lineages other than neural and potentially enable iPS to be applied in the clinical field.

The mechanobiological model of this work could also be used to compare the different nuclear mechanosensing responses in physiological vs. pathological states. For example, in cancer, it is believed that the expression of the malignant phenotype is due, at least in part, to a malfunctioning of the cell mechanoregulatory circuit. If our central hypothesis is verified, unconventional cell properties correlating the nuclear membrane structure to its permeability (including structural proteins of the cytoskeleton, the nucleus, the nuclear membrane, and the nuclear pore complexes) could become crucial new targets in cancer research.

## Author contributions

AG-G and JR: hypothesis development, numerical modeling, pre and post-processing of the numerical analysis, post-processing of the experimental STEM images, discussion and conclusions, drafting manuscript; EJ and MR: hypothesis development, experimental analysis design, pre and post-processing of the experiments, discussion and conclusions, drafting manuscript; RM: STEM imaging, writing the protocols; MT: cell culture, writing the protocols.

### Conflict of interest statement

The authors declare that the research was conducted in the absence of any commercial or financial relationships that could be construed as a potential conflict of interest.
